# Pott's Puffy Tumor Following Frontal Craniotomy: A Rare Delayed Complication in a Diabetic Elderly Woman

**DOI:** 10.1002/ccr3.70777

**Published:** 2025-08-10

**Authors:** Fereshteh Shenavaee Zare, Mohammadreza Vataniman, Soheil Shahramirad, Roya Jahanbazi, Ali Khorram

**Affiliations:** ^1^ Department of Medicine, Faculty of Medical Sciences Hormozgan University of Medical Sciences Bandar Abbas Iran; ^2^ School of Medicine Hormozgan University of Medical Sciences Bandar Abbas Iran; ^3^ Shahrood Branch Islamic Azad University Shahrood Iran; ^4^ Yazd Branch Islamic Azad University Yazd Iran

**Keywords:** craniotomy, delayed complication, diabetes, Pott puffy tumor

## Abstract

Pott's puffy tumor is a rare but serious complication that can occur years after neurosurgical procedures. In elderly diabetic patients, immunosuppression may obscure symptoms and delay diagnosis. Clinicians should maintain high suspicion when evaluating forehead swelling in such populations to ensure timely imaging and prevent intracranial complications.

## 1 Introduction

Pott's puffy tumor (PPT) is a severe complication, described by Percivall Pott in the 18th century. Pott initially described this tumor as a complication of local trauma. It is an extremely rare condition consisting of a forehead subperiosteal abscess accompanied by frontal osteomyelitis [1, 2].

PPT usually presents as a localized, tender swelling of the forehead, sometimes accompanied by fever, vomiting, headache, and periorbital swelling [1].

Clinical presentation can be misleading, especially in children and immunocompromised individuals, making urgent evaluation for possible CNS involvement essential, as intracranial complications occur in 60%–85% of cases [3]. Imaging, particularly noncontrast computed tomography (CT), is essential for early detection of frontal bone involvement and for guiding appropriate intervention. Magnetic resonance imaging (MRI) may be required to assess possible intracranial extension and associated soft tissue changes [4, 5].

The common underlying causes are frontal sinusitis, forehead trauma, insect bite, or previous surgery, and this condition occurs more frequently in children than in adults as a result of their underdeveloped frontal sinuses and increased blood flow through the diploic veins [6, 7]. The increased use of antibiotics has decreased the duration and prevalence of complications of sinusitis, making PPT a rare entity, especially in adult females [7].

In this case, we present a rare case of a 76‐year‐old diabetic female with PPT who has a history of brain mass surgery in the past 11 years. Delayed presentation of PPT more than a decade after neurosurgical intervention is exceptionally uncommon. This case illustrates the importance of maintaining long‐term clinical vigilance in patients with a history of cranial surgery, especially those with underlying immunosuppressive conditions.

## 2 Case History/Examination

**FIGURE 1:**
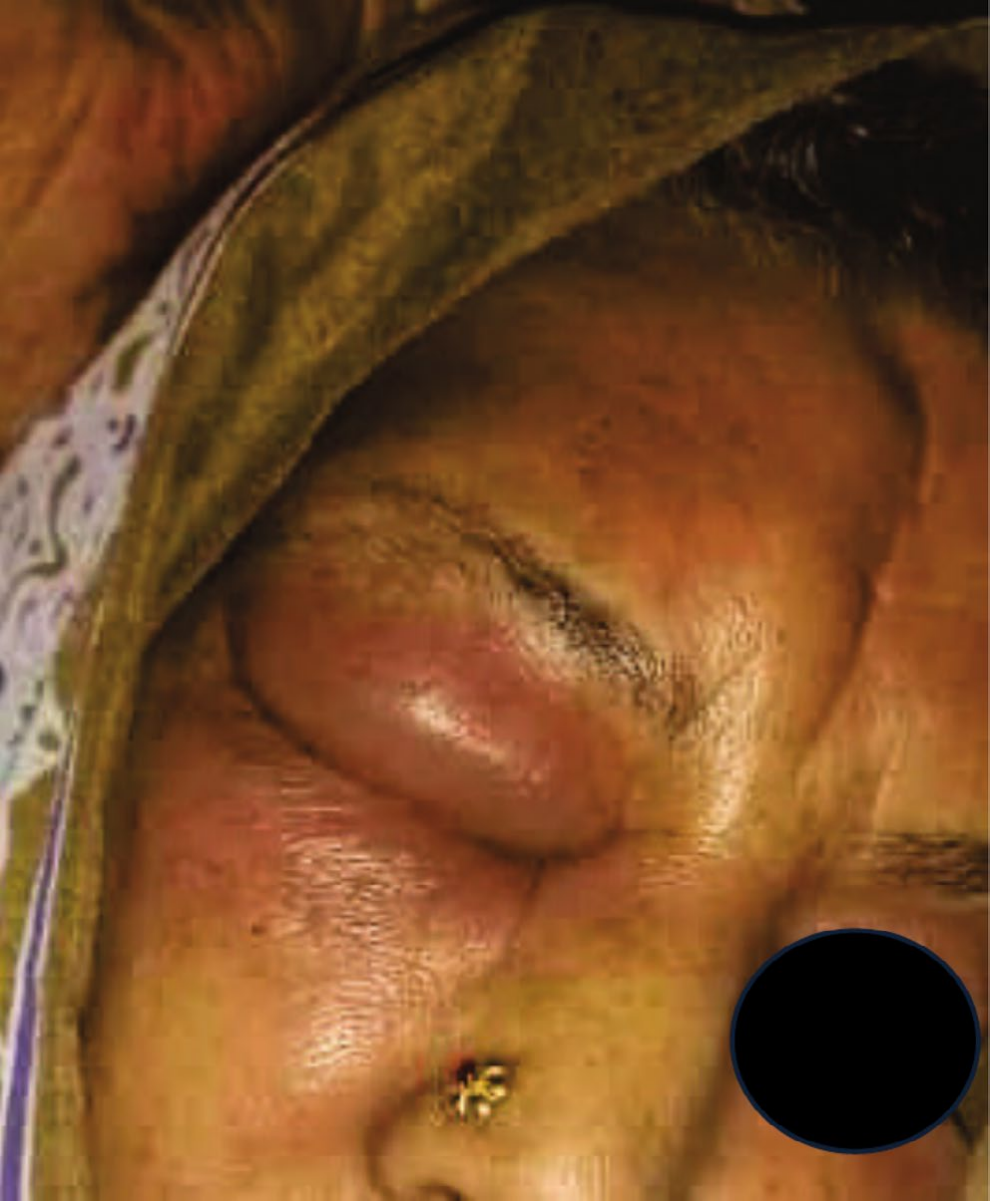
Frontal view of the patient with a tender and puffy swelling above the glabella region.

**FIGURE 2:**
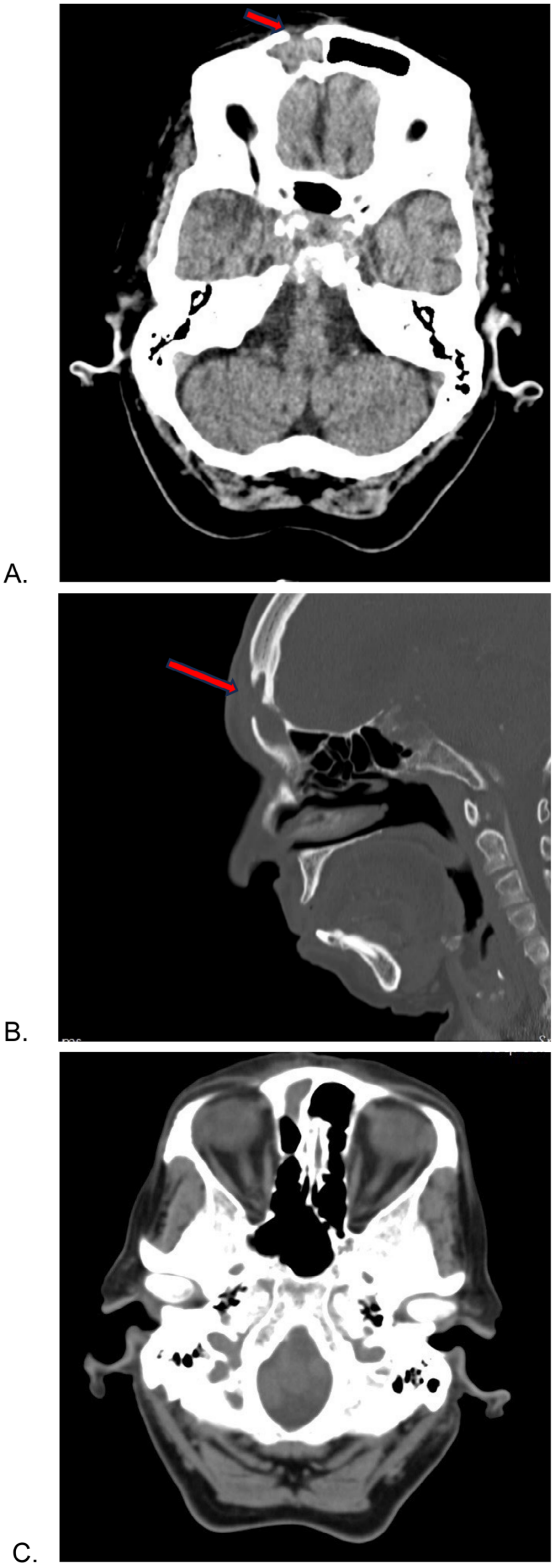
Computed tomography in patient (A–C). Right frontal sinus is completely opacified (A). A focal bony defect is visualized in the anterior wall of the right frontal sinus (arrows) consistent with prior craniotomy. There is also evidence of osteomyelitis and soft tissue swelling of the forehead. No intracranial hemorrhage, mass lesions, or radiographic evidence of cerebral infarction is identified. Opacification extends to the ethmoidal sinus (C).

**FIGURE 3:**
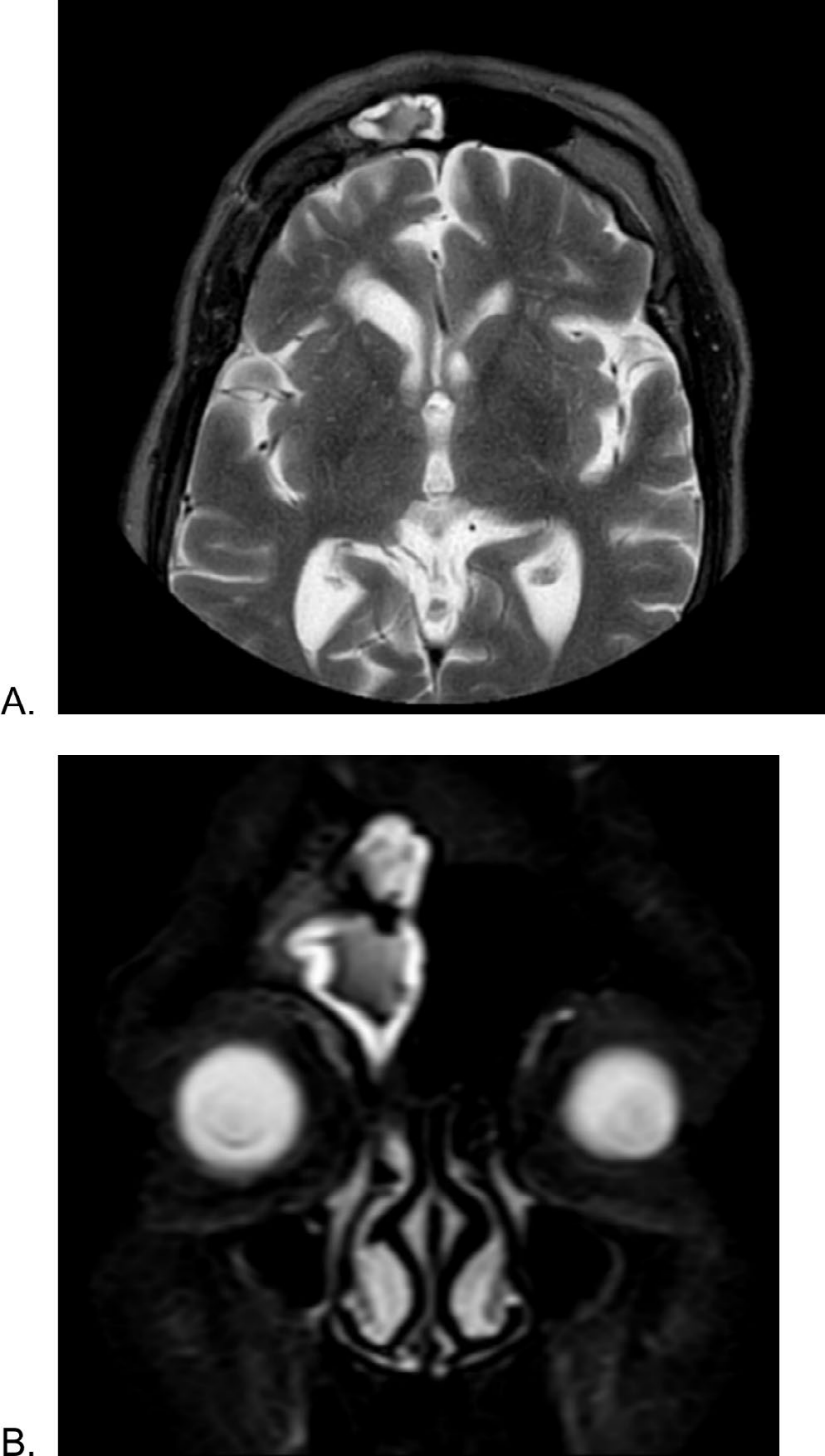
(A) Axial T2‐weighted MRI scan and (B) coronal T2‐weighted magnetic resonance imaging demonstrate opacification of the right frontal sinus, indicating fluid accumulation characteristic of sinusitis. Thinning of the anterior cortical wall is evident, which may signify osteomyelitis. Irregularities and erosions in the sinus walls could suggest a diagnosis of Pott's puffy tumor. Coronal T2‐weighted (B) reveals marked hyperintensity and expansion of the right frontal sinus. Evidence of mucosal thickening in the ethmoid sinus is also present. No parenchymal involvement is visualized. No evidence of intracranial complications is identified.

A 76‐year‐old woman had noted in the past week an enlarging, painless swelling of her right forehead. She denied any history of trauma, fever, rhinorrhea, visual problems, or vomiting in the past week. Past medical history includestype 2 diabetes mellitus, hypertension, chronic kidney disease, and one coronary angiogram. Of interest, the patient had undergone a previous frontal craniotomy for an intracranial tumor approximately 11 years prior. There were no operative records available, but the patient was able to confirm that the swelling was directly under the previous scar. On examination, the swelling was noted to be soft, fluctuant, in the right frontal region, adjacent to the scar line (Figure 1). Noncontrast computed tomography CT and MRI demonstrated an opacification in the right frontal and ethmoidal sinus (Figures 2 and 3).

On physical examination, a tender, fluctuant mass was palpated on the right forehead, extending inferiorly to involve the right upper eyelid. The overlying skin was warm to the touch, and palpation elicited pain. Notable additional swelling in the right superior orbital region was described as localized soft tissue extension.

Given the location and characteristics of the forehead swelling, initial differential diagnoses included subgaleal hematoma, frontal bone neoplasm, soft tissue abscess, cellulitis, or recurrence of a postsurgical complication.

The patient was alert and oriented. Vital signs were normal. Extraocular movements were intact, and no visual disturbances were reported. Neurological examination revealed no focal deficits. No evidence of rhinorrhea, photophobia, meningitis, or altered mental status was found. Her Glasgow Coma Scale score (GCS) was 15. Imaging demonstrated soft tissue projection in the area of the prior surgical defect. Given the clinical presentation coupled with the radiologic findings, Pott's puffy tumor could be established as the diagnosis. The orbits were unremarkable, with no evidence of intracranial hemorrhage, mass lesion, or evidence of infarction. The patient was admitted for inpatient management and started on broad‐spectrum intravenous antibiotics. Empiric therapy included clindamycin (600 mg IV every 8 h) and cefepime (1.5 g IV every 8 h). Nasal decongestants and analgesics were also provided as supportive management. During hospitalization, the patient showed significant clinical improvement with a reduction in swelling and tenderness. Purulent material was drained from the forehead at the site of the previous craniotomy scar, though no surgical intervention was immediately taken. The otorhinolaryngology team, however, scheduled endoscopic sinus surgery for definitive treatment later.

## 3 Conclusion and Results (Outcome and Follow‐Up)

Pott's puffy tumor is a rare and serious condition that requires a high index of suspicion to prevent intracranial and more severe complications, especially in older individuals, immunocompromised patients, and those with diabetes. Immunosuppression can significantly mask the typical clinical features of Pott's Puffy Tumor, leading to delays in diagnosis and increasing the risk of severe complications. Early and comprehensive imaging with CT and MRI is essential. These modalities not only help in the early detection of subperiosteal abscesses and bone involvement but are also crucial for identifying and ruling out potentially devastating intracranial complications. Pott's puffy tumor should be treated immediately upon clinical suspicion and diagnosis to prevent worsening of symptoms. Treatment includes broad‐spectrum antibiotic therapy and surgery.

## 4 Discussion

Since the description of Pott's puffy tumor in 1768, numerous cases have been reported in the literature. This complication can occur at any age, with children having the highest incidence [6]. It also affects males to a greater extent, with a 3:1 ratio [7]. Pott's puffy tumor is most commonly a localized tender swelling of the forehead caused by osteomyelitis of the frontal bone. Associated symptoms may include fever, frontal sinus tenderness, headache, purulent rhinorrhea, vomiting and nausea, and peri‐orbital swelling [8]. The differential diagnoses for this condition include hematoma, more superficial skin and soft tissue infections, soft tissue tumors, and masses (lymphoma or metastasis), preseptal cellulitis, and subgaleal hemorrhage. Differentiating PPT from these conditions can be challenging due to overlapping clinical features such as swelling, pain, and erythema. By combining clinical evaluation with imaging and laboratory findings, PPT can be accurately diagnosed and differentiated from other conditions [7]. If the infection arises through bony erosion and spreads to the intracranial cavity, especially in delayed recognition and treatment cases, it can lead to severe central nervous system (CNS) complications such as meningitis, encephalitis, epidural abscess, subdural abscess, and intracerebral abscess. These complications highlight the importance of early diagnosis and early treatment of PPT to prevent severe CNS involvement. The immediate action is high‐dose intravenous antibiotic therapy to manage the infection effectively and prevent further complications [1, 8]. Neuroimaging (CT and MRI) is essential to detect intracranial complications, which occur in approximately 72% of patients diagnosed with Pott's puffy tumor [9]. Imaging also helps delineate the extent of infection and guides surgical interventions such as debridement of the infected area and monitoring recovery [5]. CT scan is often the first imaging modality used for suspected osteomyelitis because it provides detailed visualization of the frontal bone, allowing detection of bone erosion, air–fluid levels in the sinuses, and associated findings such as subperiosteal abscess and sinus involvement [10, 11]. In this case, CT and MRI were used to assess bone tissue damage. On the patient's CT scan, there is opacification of the frontal sinus accompanied by evidence of osteomyelitis and adjacent soft tissue swelling. Intracranial complications were excluded by MRI (Figures 2 and 3). The overall imaging findings are highly suggestive of Pott's puffy tumor.

The most common causes of Pott's puffy tumor are infections secondary to sinusitis and trauma, including surgical trauma as craniotomy. Less common etiologies include odontogenic infections, cocaine abuse, wrestling injuries, insect bites, or hair transplantation [11, 12, 13]. Other noninfectious etiologies include masses such as hemangioma, lipoma, epidermal inclusion cyst, and osteoma [7]. In this case, the woman had undergone surgery on her forehead 11 years ago, and no other cause could be identified to explain the infection localized under the old scar. Microbiological diagnosis of osteomyelitis can be challenging, as cultures may be negative in up to 50% of cases; when pathogens are identified, the most common causative organisms are *Staphylococcus aureus*, *Streptococcus* species, and oral anaerobes [10].

Pott's puffy tumor can present differently in diabetic patients due to their increased susceptibility to infections. Diabetic patients might experience atypical presentations such as delayed onset [14]. In those with diabetes, localized infections and abscesses can occur decades after structural damage. Diabetes‐related complications increase susceptibility to immune suppression, infection, and impaired wound healing. Keeping blood glucose levels within normal limits is an important factor for the prevention of any complication or exacerbation of infection [2, 15]. Because of the complexity of managing infections in diabetic patients, the duration of the treatment may be longer, often requiring extended courses of antibiotics and closer monitoring for complications [15, 16].

Our patient was an elderly diabetic woman whose symptoms appeared with a delay and included a localized, tender mass. As previously mentioned, this complication is rare in the elderly and can also have a delayed onset in immunosuppressed patients. The physical examination revealed tenderness and warmth, accompanied by swelling in the upper eyelid. A complete physical examination ruled out neurological symptoms and signs of increased intracranial pressure. Treatment was initiated immediately upon diagnosis, and neuroimaging was ordered for further evaluation.

## Author Contributions

**Fereshteh Shenavaee Zare:** project administration, supervision. **Mohammadreza Vataniman:** conceptualization, supervision, writing – original draft. **Roya Jahanbazi:** conceptualization, writing – review and editing. **Soheil Shahramirad:** writing – original draft. **Ali Khorram:** conceptualization, writing – original draft.

## Ethics Statement

This case report was approved by the administration committee of ethics at Faculty of Medical Sciences, Hormozgan, Bandarabbas. The consent to participate in the study was obtained from the patient's guardian.

## Consent

Written informed consent was obtained from the patient's legal guardian for publication of this case report and any accompanying images. A copy of the written consent is available for review by the Editor‐in‐Chief of this journal.

## Conflicts of Interest

The authors declare no conflicts of interest.

## Data Availability

The authors have nothing to report.
